# Molecular Epidemiology of *Helicobacter pylori* Infection in Nepal: Specific Ancestor Root

**DOI:** 10.1371/journal.pone.0134216

**Published:** 2015-07-30

**Authors:** Muhammad Miftahussurur, Rabi Prakash Sharma, Pradeep Krishna Shrestha, Rumiko Suzuki, Tomohisa Uchida, Yoshio Yamaoka

**Affiliations:** 1 Department of Environmental and Preventive Medicine, Faculty of Medicine, Oita University, Hasama-machi, Yufu-City, Oita, Japan; 2 Gastroentero-Hepatology Division, Department of Internal Medicine, Airlangga University Faculty of Medicine, Surabaya, Indonesia; 3 Institute of Tropical Disease, Airlangga University, Surabaya, Indonesia; 4 Gastroenterology Department, Maharajgunj Medical Campus, Tribhuvan University Teaching Hospital, Kathmandu, Nepal; 5 Department of Molecular Pathology, Faculty of Medicine, Oita University, Hasama-machi, Yufu-City, Oita, Japan; 6 Department of Medicine-Gastroenterology, Baylor College of Medicine and Michael E. DeBakey Veterans Affairs Medical Center, Houston, Texas, United States of America; University of Hyderabad, INDIA

## Abstract

Prevalence of *Helicobacter pylori* infection in Nepal, a low-risk country for gastric cancer, is debatable. To our knowledge, no studies have examined *H*. *pylori* virulence factors in Nepal. We determined the prevalence of *H*. *pylori* infection by using three different tests, and the genotypes of virulence factors were determined by PCR followed by sequencing. Multilocus sequence typing was used to analyze the population structure of the Nepalese strains. The prevalence of *H*. *pylori* infection in dyspeptic patients was 38.4% (56/146), and was significantly related with source of drinking water. In total, 51 strains were isolated and all were *cagA*-positive. Western-type-*cagA* (94.1%), *cagA* pre-EPIYA type with no deletion (92.2%), *vacA* s1a (74.5%), and m1c (54.9%) were the predominant genotypes. Antral mucosal atrophy levels were significantly higher in patients infected with *vacA* s1 than in those infected with s2 genotypes (P = 0.03). Several Nepalese strains were *H*. *pylori* recombinants with genetic features of South Asian and East Asian genotypes. These included all East-Asian-type-*cagA* strains, with significantly lesser activity and inflammation in the corpus than the strains of the specific South Asian genotype (P = 0.03 and P = 0.005, respectively). Although the population structure confirmed that most Nepalese strains belonged to the hpAsia2 population, some strains shared hpEurope- and Nepalese-specific components. Nepalese patients infected with strains belonging to hpEurope showed higher inflammation in the antrum than strains from the Nepalese specific population (P = 0.05). These results support that ancestor roots of Kathmandu`s people not only connected with India alone.

## Introduction

*Helicobacter pylori*, a major pathogen of the gastrointestinal tract, has been implicated in a wide spectrum of gastric disorders, including gastritis, peptic ulcer, gastric cancer, and mucosa-associated lymphoid tissue lymphoma [1,2]. However, the infection remains latent in the majority of infected patients, and only a minority of individuals with *H*. *pylori* infection ever develop the disease [3]. Although *H*. *pylori* infection is a major factor in the development of gastric cancer [4], the difference in *H*. *pylori* infection rate between countries is not enough to explain the difference in the incidence of gastric cancer in the world. In addition to host and environmental factors, in part, the difference in the incidence of gastric cancer irrespective of *H*. *pylori* infection rate can be explained by the difference of virulence factors [5].

The *cagA* gene, which encodes a highly immunogenic protein (CagA), is the most extensively studied *H*. *pylori* virulence factor. CagA has been reported to interact with various target molecules in host cells; of these, the best-studied molecule is the cytoplasmic Src homology 2 domain of Src homology 2 phosphatase (SHP-2) [6]. Recently, sequences of CagA have been annotated according to segments (20–50 amino acids) flanking the Glu-Pro-Ile-Tyr-Ala (EPIYA) motifs (i.e., segments EPIYA-A,-B,-C, or-D) [7]. Western-type-*cagA* strains are reported to be less virulent than their Eastern counterparts. The pre-EPIYA region, located about 300 base pairs (bp) upstream of the first EPIYA motif, has also been investigated as a virulence factor. Alignment of these sequences revealed that a 39 bp deletion was present in most strains isolated from East Asia, but was absent in most strains from Western countries (non-deletion type) [8].

In the applicable of markers of genomic diversity, *vacA* is the second most extensively studied *H*. *pylori* virulence factor. The differences in the *vacA* structure at the signal region (s1 and s2) and the middle region (m1 and m2) lead to variations in the vacuolating activity of different *H*. *pylori* strains. The *vacA* s1 and m1 types are subdivided into s1a, s1b, and s1c, and m1a, m1b, and m1c, respectively [9]. The *vacA* s2 genotype encodes a shorter extension of the N-terminal peptide on the mature protein, which blocks the vacuolating activity. Conversely, infection with *vacA* s1 strains has been linked to gastric inflammation and duodenal ulceration with enhanced cytotoxin activity. In general, the *vacA* s1m1 strains produce a large amount of toxin with high vacuolating activity in gastric epithelial cells, while s1m2 strains produce moderate amounts of toxin, and s2m2 strains produce toxin rarely or not at all [10].

Genetic studies have established that *H*. *pylori* is highly diverse, with the diversity being influenced by both geography and human ethnicity. Genetic diversity within *H*. *pylori* populations also tends to decrease with increasing distance from Africa, consistent with a similar but stronger cline observed in humans [11,12]. Multilocus sequence typing (MLST) of seven housekeeping genes from several hundred *H*. *pylori* strains isolated from different geographical, ethnic, and/or linguistic origins showed that *H*. *pylori* followed human migration out of Africa and identified seven *H*. *pylori* populations, which are designated as hpAfrica1, hpAfrica2, hpNEAfrica, hpEurope, hpEastAsia, hpAsia2, and hpSahul [11–13]. In addition, the distribution of gastric cancer incidence seems to be closely related to these *H*. *pylori* groups. A high incidence of gastric cancer was found in regions with more prevalent hpEastAsia strains (especially hspEAsia) [14]. On the other hand, the incidence of gastric cancer is very low in Africa, where most strains are hpNEAfrica, hpAfrica1, or hpAfrica2, and in South Asia, where most strains are hpAsia2. Overall, the African and Asian enigmas might be explained, at least in part, by the different genotypes of *H*. *pylori* circulating in these different geographic areas [15].

Nepal is a small landlocked country in South Asia, located in the Himalayas. Kathmandu is the capital and the largest urban agglomerate of Nepal. The age-standardized incidence rate (ASR) of gastric cancer in Nepal is reported to be 5.3 cases per 100,000 population per year, which is similar to that of neighboring countries such as India and Bangladesh, and is much lower than Bhutan and China (6.1, 5.8, 17.2, and 22.7/100,000, respectively) (available from the International Agency for Research on Cancer, GLOBOCAN 2012; http://globocan.iarc.fr). The physical and cultural landscape of Nepal can be divided into three distinct regions. The mountainous region in the north is culturally linked to the Buddhists of Tibet. Terai, the southernmost region of Nepal, is culturally linked to North India. Between these regions are the majority of the Nepalese population, whose cultural practices incorporate both Buddhist and Hindu traditions. Genetic analyses have revealed that the Kathmandu ancestry is a combination of East and South Central Asian lineages [16].

Although the prevalence of *H*. *pylori* infections in Nepal has been investigated, the results have varied, with prevalence ranging from 16.3% to 70.5% [17,18]. In addition, to our knowledge, no report has examined *H*. *pylori* virulence factors in Nepalese strains. We hypothesized that the *H*. *pylori* genotype and associated virulence factors contribute to the low incidence of gastric cancer in Nepal. In this study, we examined the prevalence of *H*. *pylori* infection in Nepal using three different tests and analyzed the virulence factors in Nepalese strains. Furthermore, we evaluated human migration patterns using *H*. *pylori* as a tool.

## Materials and Methods

### Study population

We enrolled 146 volunteers with dyspeptic symptoms (76 women and 70 men; mean age of 42.2 ± 15.7 years) consecutively from July 2012 to September 2012. The survey was conducted at the endoscopy services section of the Gastroenterology Department, Tribhuvan University Teaching Hospital (TUTH), Kathmandu, Nepal. Patients with upper gastrointestinal bleeding and history of partial gastric resection, were excluded from this study. Written informed consent was obtained from all participants, and the study protocol was approved by the Ethics Committee of TUTH and Oita University Faculty of Medicine, Japan. We also obtained information about medications (antibiotics, histamine-2 receptor antagonists, or proton pump inhibitors), and smoking and alcohol habits. To minimize potential bias, the expert pathologist (TU) as had performed histological examinations for studies in Bhutan and Indonesia [19,20] evaluated all of the histological results in this study. Experienced endoscopists collected four gastric biopsy specimens during each endoscopy session: three samples from the lesser curvature of the antrum approximately 3 cm from the pyloric ring, and one sample from the greater curvature of the corpus. Biopsy specimens for culture were immediately placed at −20°C, and stored at −80°C within a day of collection until used for culture testing. With dry ice the specimens were sent to Japan and performed culture in the same day when the specimens arrive in Japan. Three antrum specimens were used for *H*. *pylori* culture, rapid urease test (Campylobacter-like organism [CLO] test, Kimberly-Clark Ballard Medical Products, Roswell, GA, USA), and for histological examination. One corporal specimen was also used for histological examination. Peptic ulcers and erosive gastritis were identified by endoscopy.

### Status of *H*. *pylori* infection

To maximize the diagnostic accuracy, we used three different methods, including culture, histology confirmed by immunohistochemistry (IHC), and the rapid urease test.

For *H*. *pylori* culture, one antral biopsy specimen was homogenized in saline and inoculated onto Mueller Hinton II Agar medium (Becton Dickinson, NJ, USA) supplemented with 7% horse blood without antibiotics. The plates were incubated for up to 10 days at 37°C under microaerophilic conditions (10% O_2_, 5% CO_2_, and 85% N_2_). *H*. *pylori* isolates were identified on the basis of colony morphology, Gram staining results, and positive reactions for oxidase, catalase, and urease. Isolated strains were stored at −80°C in Brucella Broth (Difco, NJ, USA) containing 10% dimethyl sulfoxide and 10% horse serum.

### Determination of gastritis stage

All biopsy materials for histological testing were fixed in 10% buffered formalin and embedded in paraffin. Serial sections were stained with hematoxylin and eosin as well as May–Giemsa stain. The degree of inflammation, neutrophil activity, atrophy, intestinal metaplasia, and bacterial density were classified into four grades according to the updated Sydney system: 0, normal; 1, mild; 2, moderate; and 3, marked [21]. Samples with grade 1 or more atrophy were considered atrophy-positive [22]. In addition, the gastritis stage was assessed based on topographic locations (antrum and corpus), according to the Operative Link on Gastritis Assessment (OLGA) system [23].

### Immunohistochemistry

Immunohistochemistry was performed as previously described [24]. Briefly, after antigen retrieval and inactivation of endogenous peroxidase activity, tissue sections were incubated with anti-α-*H*. *pylori* antibody (DAKO, Denmark) overnight at 4°C. After washing, the sections were incubated with biotinylated goat anti-rabbit IgG (Nichirei Co., Japan), followed by incubation with an avidin-conjugated horseradish peroxidase solution (Vectastain Elite ABC Kit; Vector Laboratories Inc., Burlingame, CA, USA). Peroxidase activity was detected using an H_2_O_2_/diaminobenzidine substrate solution.

### *H*. *pylori* isolation and genotyping

*H*. *pylori* colonies were cultured from antral biopsy specimens using standard methods [10]. *H*. *pylori* DNA was extracted from these colonies for *H*. *pylori* genotyping using the QIAamp DNA Mini Kit (QIAGEN, Valencia, CA) according to the manufacturer directions. The *cagA* status was determined by PCR amplification and direct sequencing of the EPIYA repeat and the pre-EPIYA regions as described previously [8,25]. The *vacA* genotyping (s1a, s1b, s1c, or s2; and m1a, m1b, m1c, or m2) was performed as described previously [9,26,27]. A phylogenetic tree was constructed using reference strains of the *cagA* 3´ repeat region and *vacA* m regions from GenBank that were integrated with our Nepal sequence data. DNA sequencing was performed using a Big Dye Terminator v3.1 Cycle Sequencing Kit on an AB 3130 Genetic Analyzer (Applied Biosystems, Foster City, CA) according to the manufacturer instructions. Multiple sequence alignments of the *cagA* pre-EPIYA, *cagA*, and *vacA* sequences were generated using the MAFFT version 7 (available in http://mafft.cbrc.jp/alignment/server/) and confirmed by visual inspection.

### Population structure analysis of *H*. *pylori* strains

We analyzed bacterial population structure using STRUCTURE (v.2.3.2) software [28]. Markov Chain Monte Carlo (MCMC) simulations of STRUCTURE were run in the admixture model with a burn-in of 20,000, followed by 30,000 iterations for each run. The number of tentative populations (K) was set from 7 to 15, and 5 runs were executed for each K.

### Data analysis

Discrete variables were tested using the chi-square test; continuous variables were tested using the Mann-Whitney *U* and *t*-tests. A multivariate logistic regression model was used to calculate the odds ratios (OR) of the clinical outcomes that included age, sex, *H*. *pylori* infection status, demographic, sanitation type, sociocultural factors, and gastritis type. All determinants with P values of < 0.10 were entered together into the full logistic regression model, and the model was reduced by excluding variables with P values of > 0.10. The OR and 95% confidence interval (CI) were used to estimate the risk. A P value of < 0.05 was accepted as statistically significant. The SPSS statistical software package version 18.0 (SPSS, Inc., Chicago, IL) was used for all statistical analyses.

## Results

### *H*. *pylori* infection rate in dyspeptic patients in Kathmandu

Histology confirmed by immunohistochemistry showed the highest prevalence of *H*. *pylori* infection (37.7%, 55/146), whereas using culture it was 34.9% (51/146). Using histology confirmed IHC as a gold standard; the sensitivity, specificity, negative predictive value (NPV) and positive predictive value (PPV) of culture were 92.7%, 100.0%, 95.8% and 100.0%, respectively. Overall accuracy rates were 97.3%. Forty of 146 (27.4%) study participants were positive for *H*. *pylori* by all three tests. However when patients were categorized as positive for *H*. *pylori* infection with at least one positive test result, the overall *H*. *pylori* infection rate was 38.4% (56/146).

The adjusted OR were calculated for *H*. *pylori* infection (Table 1). Women showed a significantly higher *H*. *pylori* infection rate than did the men. The prevalence of *H*. *pylori* infection significantly lower among people who used tap water as source of drinking water. However, there were no statistically significant relationships between the *H*. *pylori* infection rate and ethnicity education level, religion, occupation, marital status, family members, type of latrine, history of drugs, smoking habit, and alcohol consumption. In the subsequent analysis, patients were considered to be negative for *H*. *pylori* infection when all three test results were negative, whereas patients with at least one positive test result were considered positive for *H*. *pylori* infection.

**Table 1:** Association of demographic, sanitation and sociocultural factors with *H*. *pylori* infection status.

Variable	Total (+*H*. *pylori*%)	Crude OR	95% CI for OR	P
Age				
≤29	32 (37.5%)	1.26	0.46–3.46	0.66
30–39	34 (32.4%)	1.00		
40–49	34 (38.2%)	1.29	0.48–3.51	0.61
50–59	25 (48.0%)	1.93	0.67–5.59	0.23
≥60	21 (38.1%)	1.29	0.41–4.01	0.66
Gender				
Males	70 (30.0%)	1.00		
Females	76 (46.1%)	1.99	1.01–3.94	0.05
Religion				
Hinduism	118 (38.1%)	1.00		
Buddhism	28 (39.3%)	1.05	0.45–2.44	0.91
Ethnic				
Madhesi	4 (25.0%)	1.00		
Brahmin	51 (27.5%)	1.14	0.11–11.85	0.92
Chhetri	26 (53.8%)	3.50	0.32–38.23	0.30
Mongols	33 (42.4%)	2.21	0.21–23.56	0.51
Others	32 (40.6%)	2.05	0.19–21.97	0.55
Education				
Illiterate	47 (53.2%)	1.67	0.38–7.32	0.50
Literate	33 (33.3%)	3.79	0.92–15.54	0.06
Elementary school	13 (23.1%)	1.00		
High school	39 (33.3%)	1.67	0.39–7.12	0.49
Scholars	14 (28.6%)	1.33	0.24–7.56	0.75
Occupation				
Government job	8 (25.0%)	1.00		
Business	24 (33.3%)	1.50	0.25–9.18	0.66
Farmers	34 (50.0%)	3.00	0.53–17.02	0.22
Housewife	37 (35.1%)	1.63	0.29–9.23	0.58
Student	6 (42.9%)	2.25	0.33–15.33	0.41
Private job	13 (30.8%)	1.33	0.18–9.73	0.78
Unemployed	16 (37.5%)	1.88	0.27–11.96	0.54
Marital status				
Unmarried	16 (37.5%)	1.00		
Married	130 (38.5%)	1.04	0.36–3.04	0.94
Food habit				
Regular	65 (35.4%)	1.00		
Irregular	81 (40.7%)	1.26	0.64–2.46	0.51
Family members				
Crowded	46 (34.8%)	1.00		
Uncrowded	100 (40.0%)	1.25	0.60–2.59	0.55
Source of drinking water				
Tap water	124 (35.5%)	1.00		
Wells	10 (40.0%)	1.21	0.33–4.53	0.78
Spring	12 (66.7%)	3.64	1.04–12.76	0.04
Latrine				
Private	94 (36.2%)	1.00		
Public	52 (42.3%)	1.29	0.65–2.59	0.47
History of drugs (PPI, H2blockers, antibiotics)				
No	40 (37.5%)	1.00		
Yes	106 (38.7%)	1.05	0.50–2.23	0.90
Smokers				
Yes	39 (33.3%)	1.00		
No	107 (40.2%)	1.34	0.62–2.90	0.45
Alcohol consumption				
No	119 (37.0%)	1.00		
Yes	27 (44.4%)	1.36	0.59–3.18	0.47

Among 146 patients, 44 (30.1%) showed no activity or inflammation in either the antrum or the corpus by histological examination; these patients were considered to be the normal group. Among 87 patients with histological gastritis, 46 (52.9%) were positive for *H*. *pylori*, a significantly higher rate than that in the normal group (P < 0.001). Histological findings in people from Kathmandu showed that 92 patients (63.0%) had mucosal atrophy in the antrum, and 14 (9.6%) patients also had corporal mucosal atrophy. Histological scores according to *H*. *pylori* infection status are shown in Table 2. As expected, all histological scores were significantly higher in patients positive for *H*. *pylori* than in patients negative for *H*. *pylori*.

**Table 2:** Histological scores according to H. pylori infection status by culture.

	*H*. *pylori* (+)	*H*. *pylori* (-)	P
N	51	95	
**Antrum**			
Activity	1.39 (1)	0.25 (1)	<0.0001
Inflammation	1.71 (2)	0.61 (1)	<0.0001
Atrophy	1.20 (1)	0.50 (0)	<0.0001
Intestinal metaplasia	0.08 (0)	0.09 (0)	0.89
Bacterial density	1.45 (1)	0.02 (0)	<0.0001
**Corpus**			
Activity	1.04 (1)	0.20 (0)	<0.0001
Inflammation	0.88 (1)	0.25 (0)	<0.0001
Atrophy	0.14 (0)	0.11 (0)	0.54
Intestinal metaplasia	0.06 (0)	0.01 (0)	0.97
Bacterial density	1.47 (1)	0.04 (0)	<0.0001
**OLGA score**	1.20 (1)	0.50 (0)	<0.0001

### Genotypes

A total of 51 *H*. *pylori* strains were isolated, from 19 men (age range, 17–77 years; mean age, 41.7 years) and 32 women (age range, 17–69 years; mean age, 43.5 years). The strains consisted of 41 from patients with gastritis, 2 with gastric ulcer (GU), 5 with duodenal ulcer (DU), and 3 with gastric cancer (Table 3). The average age was significantly higher in gastric cancer patients than in patients with gastritis (P < 0.03).

**Table 3:** Association between *H*. *pylori* virulence factors and clinical outcomes.

	Total		Gastritis		Duodenal ulcer		Gastric ulcer		Gastric cancer	
Description of genotype	No	%	No	%	No	%	No	%	No	%
Total studied	51		41		5		2		3	
Mean age (yr)	42.8±15.5		41.2±14.2		41.6±18.8		45±14.1		66.3±15.9	
Female	32	62.7%	27	65.9%	2	40.0%	2	100.0%	1	33.3%
East-Asian-type-*cagA*	3	5.9%	3	7.3%	0	0.0%	0	0.0%	0	0.0%
Western-type-*cagA*	48	94.1%	38	92.7%	5	100.0%	2	100.0%	3	100.0%
Non deletion type	47	92.2%	37	90.2%	5	100.0%	2	100.0%	3	100.0%
6 bp deletion type	3	5.9%	3	7.3%	0	0.0%	0	0.0%	0	0.0%
18 bp deletion type	1	2.0%	1	2.4%	0	0.0%	0	0.0%	0	0.0%
*vacA* s1	49	96.1%	39	95.1%	5	100.0%	2	100.0%	3	100.0%
*vacA*s2	2	3.9%	2	4.9%	0	0.0%	0	0.0%	0	0.0%
*vacA* m1	31	60.8%	25	61.0%	5	100.0%	0	0.0%	1	33.3%
*vacA* m2	20	39.2%	16	39.0%	0	0.0%	2	100.0%	2	66.7%
*vacA* s1a	38	74.5%	30	73.2%	4	80.0%	1	50.0%	3	100.0%
*vacA* s1c	5	9.8%	4	9.8%	0	0.0%	1	50.0%	0	0.0%
Mixed *vacA* s1*	6	11.8%	5	12.2%	1	20.0%	0	0.0%	0	0.0%
*vacA* m1b	3	5.9%	3	7.3%	0	0.0%	0	0.0%	0	0.0%
*vacA* m1c	28	54.9%	22	53.7%	5	100.0%	0	0.0%	1	33.3%
*vacA* s1m1	31	60.8%	25	61.0%	5	100.0%	0	0.0%	1	33.3%
*vacA* s1m2	18	35.3%	14	34.1%	0	0.0%	2	100.0%	2	66.7%
*vacA* s2m2	2	3.9%	2	4.9%	0	0.0%	0	0.0%	0	0.0%
*vacA* s1am1b	2	3.9%	2	4.9%	0	0.0%	0	0.0%	0	0.0%
*vacA* s1am1c	25	49.0%	20	48.8%	4	80.0%	0	0.0%	1	33.3%
*vacA* s1am2	11	21.6%	8	19.5%	1	20.0%	0	0.0%	2	66.7%
*vacA* s1cm1b	1	2.0%	1	2.4%	0	0.0%	0	0.0%	0	0.0%
*vacA* s1cm1c	1	2.0%	1	2.4%	0	0.0%	0	0.0%	0	0.0%
*vacA* s1cm2	3	5.9%	2	4.9%	1	20.0%	0	0.0%	0	0.0%
*vacA* mix s1- m1c	2	3.9%	1	2.4%	0	0.0%	1	50.0%	0	0.0%
*vacA* mix m2	4	7.8%	4	9.8%	0	0.0%	0	0.0%	0	0.0%
Western-type-*cagA/vacA* s1m1	29	56.9%	23	56.1%	5	100.0%	0	0.0%	1	33.3%
Western-type-*cagA/vacA* s1m2	16	31.4%	13	31.7%	0	0.0%	2	100.0%	1	33.3%
Western-type-cagA/vacA s2m2	1	2.0%	1	2.4%	0	0.0%	0	0.0%	0	0.0%
Western-type-*cagA/vacA* s1am1b	2	3.9%	2	4.9%	0	0.0%	0	0.0%	0	0.0%
Western-type-*cagA/vacA* s1am1c	25	49.0%	20	48.8%	4	80.0%	0	0.0%	1	33.3%
Western-type-*cagA/vacA* s1am2	2	3.9%	0	0.0%	0	0.0%	0	0.0%	2	66.7%

* Mixed *vacA* s1 is a strain had double of subtype of *vacA* s1

**Fig 1:**
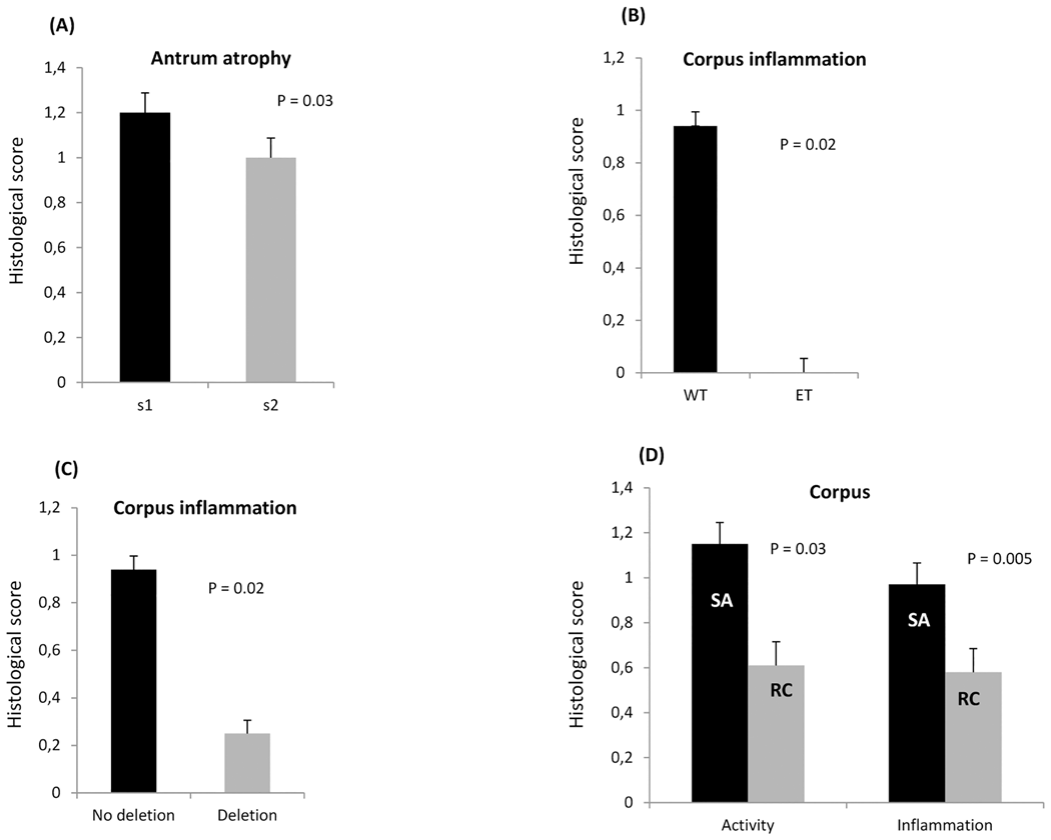
The association of histological findings and genotypes in *Helicobacter pylori* Nepalese strains. (A) The *vacA* s1 genotype had significantly greater antrum mucosal atrophy than the *vacA* s2 genotype. Inflammation in the corpus was more highly associated with the Western-type-*cagA* than East-Asian-type-*cagA* type (B), and with the non-deletion type than the deletion type (C). The specific South Asian genotype had higher levels of activity and inflammation in the corpus than the recombination genotype (D).

All strains possessed the *cagA* gene. Sequence analyses revealed that 44 strains were the ABC type (86.3%), two were AB type, one was ACC type, and one was BC type. All of the strains were considered to be Western-type-*cagA* (48/51, 94.1%). East-Asian-type-*cagA* (ABD) was found in only 5.9% (3/51) of isolates, which were from participants of three different ethnic groups (Chhetri, Mongols, and others). Sequence analyses of a region 300 bp upstream of the first EPIYA motif revealed that 92.2% (47/51) of strains were the non-deletion type (mostly observed in Western countries) and 5.9% (3/51) of strains were the 6 bp deletion type. The remaining one strain was the 18 bp deletion type, which is typically observed in Vietnamese strains [8], and none were the 39 bp deletion type. All strains of the pre-EPIYA non-deletion type were Western-type-*cagA*, and, conversely, only one Western-type-*cagA* was not classified as a non-deletion type. On the other hand, as for East-Asian-type-*cagA* strains, one was the 18 bp deletion type and two were the 6 bp deletion type. Histological analysis showed that inflammation in the corpus was significantly greater in Western-type-*cagA* than in East-Asian-type-*cagA* (mean [median]; 0.94 [1] vs. 0.00 [0]; P = 0.02) (Fig 1B), which is in contrast with the current consensus on pathology associated with these *H*. *pylori* types. Inflammation in the corpus was also significantly greater with the non-deletion type than in the deletion type (mean [median]; 0.94 [1] vs. 0.25 [0]; P = 0.02) (Fig 1C).

**Fig 2:**
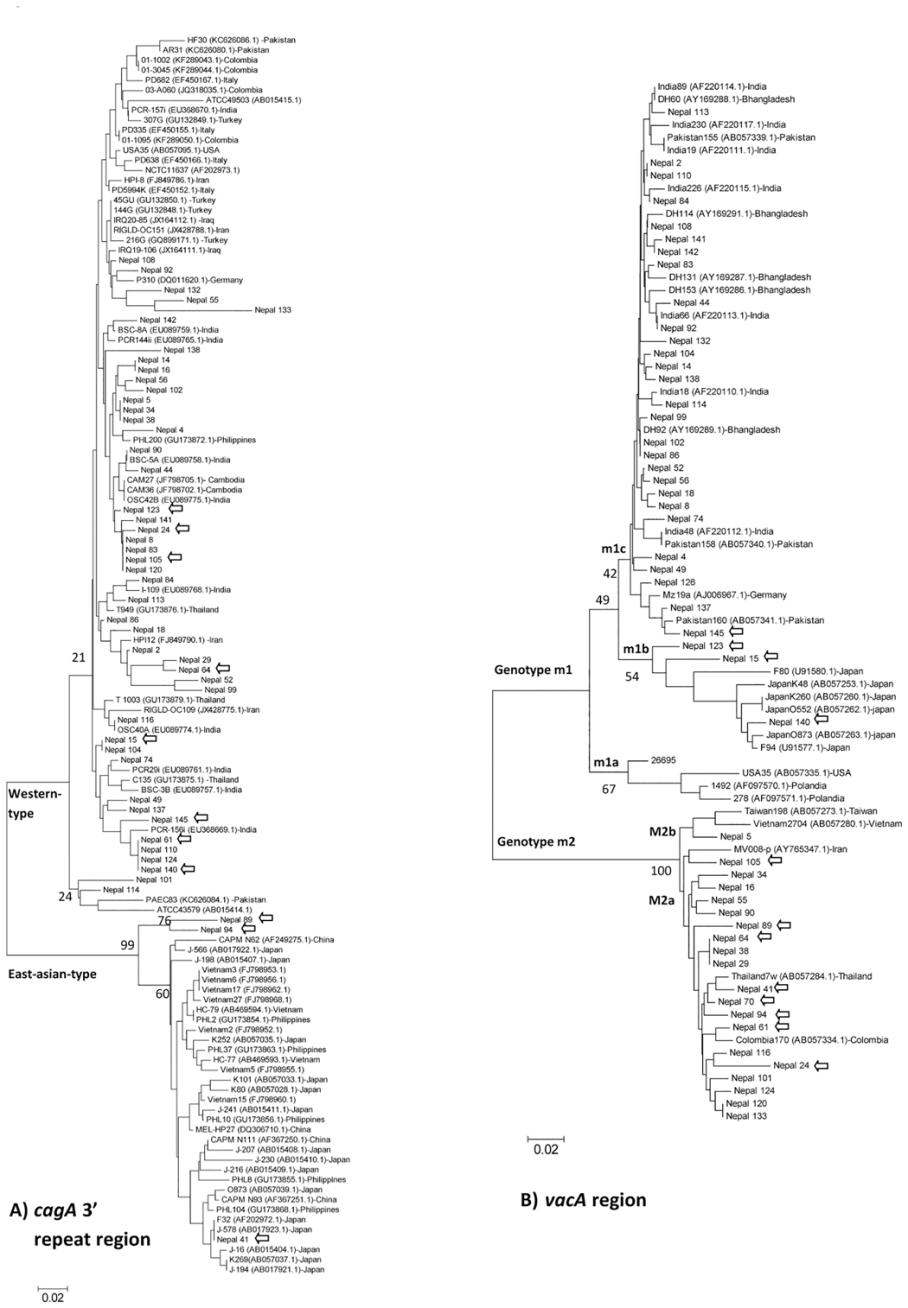
Phylogenetic analysis of the *cagA* 3´ repeat region (A), *vacA* m region (B), and reference sequences of *Helicobacter pylori*. Genetic distances were estimated by the six-parameter method and phylogenetic trees were constructed by the neighbor-joining method. Strains with GenBank accession numbers are reference strains. Bootstrap values are shown along each main branch. The lengths of the horizontal bars indicate the number of nucleotide substitutions per site.

For *vacA*, the predominant genotype was s1 (49/51, 96.1%), consisting of 74.5% (38/51) of the s1a genotype, which is typical of South Asia strains, 9.8% (5/51) of the s1c genotype that is predominant in East Asia, 11.8% (6/51) of the multiple *vacA* s1a-s1b and s1a-s1c (mix s1) genotype, and no strains of the western type *vacA* genotype (s1b). The *vacA* s2 genotype was found in only 3.9% (2/51) of isolates. The prevalence of the *vacA* m1 genotype was 60.8% (31/51), and the *vacA* m2 genotype was found in 39.2% (20/51) of isolates. Sequence analysis of the 0.7-kb middle region of *vacA* was consistent with the PCR results, and indicated a clear separation between m1 and m2 sequences (Fig 2). The m1c, a typical South Asia genotype were predominant (28/32, 87.5%), while 4/32 (12.5%) were infected with m1b, a genotype mostly observed in East Asian strains, and none had m1a. For the combined *vacA* s and m regions, the predominant types were s1a-m1c (49%, 25/51) and s1a-m2 (21.6%, 11/52). Although there was no relationship between *cagA*, pre-EPIYA types, and *vacA* s and m types with clinical outcomes in the Nepalese population (P = 0.86, P = 0.96, P = 0.92 and P = 0.06, respectively), all strains from DU, GU, and gastric cancer cases were of the Western-type-*cagA*, non-deletion type, and *vacA* s1. All strains from DU cases were m1c, and all strains from GU were m2. Histological findings of the *vacA* s genotypes showed that s1 isolates were more highly associated with antral mucosal atrophy than s2 genotypes (mean [median]; 1.20 [1] vs. 1.00 [1], P = 0.03) (Fig 1A). Even after adjustment by age and sex in the multivariate analysis, atrophy scores in the antrum were significantly higher in patients with s1 than those with s2 genotypes (OR = 30.4; 95% CI: 0.97–95.1; P = 0.05).

The phylogenetic tree of the complete amino acid sequences of the middle region *vacA* demonstrated the genetic relationships among the Nepalese strains and reference strains (Fig 2). Western-type-*cagA* sequences from this study showed a clear separation from East-Asian-type-*cagA* sequences. The majority of Western-type-*cagA* Nepalese strains were closely related to those of South and Southeast Asian countries. Phylogenetic analysis of the *vacA* m region showed that only one strain (Nepal 5) clustered with 100% bootstrap support with the East-Asian-type m2 (m2b) strains (Fig 2B). It has been suggested that the *vacA* m2 genotype can be divided into m2a (both East Asian and non-Asian) and m2b (East Asian) [9]. This result indicated that the m2 genotype in the Nepalese strains were concordant with the non-East Asian type m2 strains. In addition, m1c Nepalese strains were closely related to reference strains from South Asian countries, including India and Bangladesh.

Despite this support for the previously established *H*. pylori phylogeny, we found many inconsistencies in the genotypes of these Nepalese strains, which may reflect the recombination of *H*. *pylori* genetic features of South Asian and East Asian genotypes. For example, strain Nepal 15 was of the Western-type-*cagA* and pre-EPIYA non-deletion type (specific for the South Asian genotype); however, the *vacA* genotypes were s1c and m1b (specific for the East Asian genotype). Table 4 shows Nepalese strains categorized into two groups; specific South Asian genotypes (s1a, m1c/m2, Western-type-*cagA*, and the non-deletion type) is the predominant group and the recombinant genotype (also showed by arrows in Fig 2) is the other group. Interestingly, all East-Asian-type-*cagA* strains included a recombinant genotype. Fig 2A shows that although strain Nepal 41 was found to be closely related to typical East-Asian-type-*cagA* strains, two strains (Nepal 89 and 94) were distinct (bootstrap 99%). Moreover, all East-Asian-type-*cagA* strains were m2 and pre-EPIYA non-39 bp deletion types (most East-Asian type-*cagA* strains were m1 and pre-EPIYA 39 bp deletion types [5,8]. Interestingly, this specific South Asian genotype showed significantly greater activity and inflammation in the corpus than the recombination genotypes (mean [median]; 1.15 [1] vs. 0.61 [1]; P = 0.03; 0.97 [1] vs. 0.58 [1]; P = 0.005) (Fig 1D).

**Table 4:** Analysis *vacA*, *cagA* repeat and pre-EPIYA genotypes of Nepalese *H*. *pylori* strains.

Strain	*vacA* s	*vacA* m	*cagA* repeat(EPIYA segments)	Pre-EPIYA	Diagnosis
**Specific South Asian genotype**					
27 Nepalese strains	s1a	m1c	Western type	No deletion type	21 gastritis, 5 DU, 1 cancer
11 Nepalese strains	s1a	m2	Western type	No deletion type	8 gastritis, 1 GU, 2 cancer
Nepal 124	s2	m2	Western type	No deletion type	1 gastritis
**Recombination of the South Asian and East Asian genotype (?)**					
Nepal 24, 105	s1c	m2	Western type	No deletion type	1 GU, 1 Gastritis
Nepal 61	s1c	m2	Western type	6 bp deletion type	gastritis
Nepal 15	s1c	m1b	Western type	No deletion type	gastritis
Nepal 145	s1c	m1c	Western type	No deletion type	gastritis
Nepal 123, 140	s1a	m1b	Western type	No deletion type	2 gastritis
Nepal 41	s2	m2	East-Asian type	6 bp deletion type	1 Gastritis
Nepal 89	s1a	m2	East-Asian type	6 bp deletion type	1 Gastritis
Nepal 64, 70	s1c	m2	Western type	No deletion type	2 gastritis
Nepal 94 (Specific East Asian genotype?)	s1c	m2	East-Asian type	18 bp deletion type	gastritis

### Population structure

**Fig 3:**
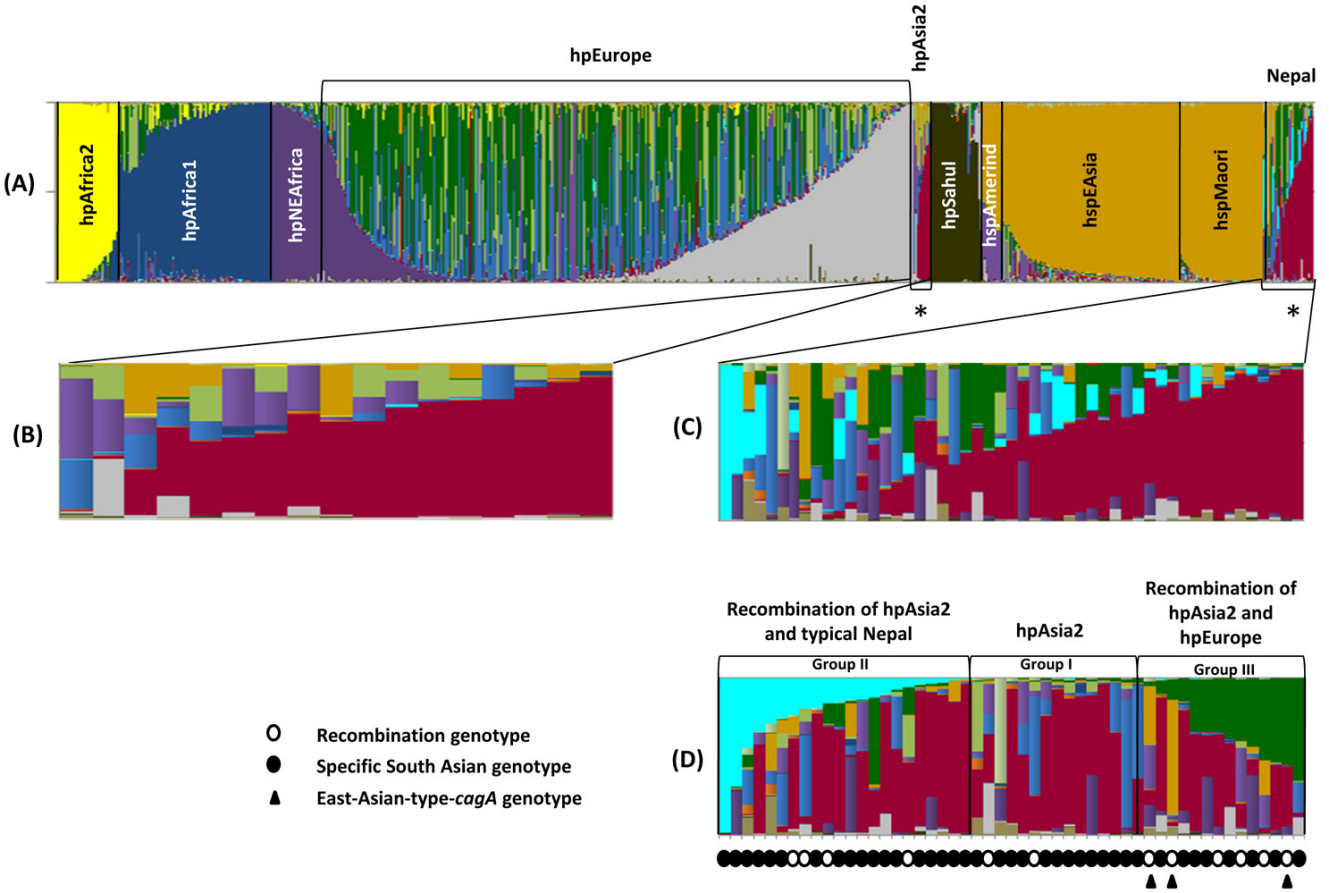
Population structure of Nepalese strains. Results of the population analysis by STRUCTURE with K, or a tentative number of populations, set to 15. Each vertical bar represents one sample. Colors indicate the population components and the lengths of the colors in the vertical bars are proportional to the probability that the sample belongs to the population of that color. The order of the samples is the same in all the bar charts. The stars in panel A represent Nepalese strains and hpAsia2 strains that share the same population component. The bars were aligned from left to right in descending order of red components (C), and navy blue and dark green components (D). The recombination genotype and East-Asian-type-*cagA* strains are marked below the bar chart (D).

To investigate the population structure of the Nepalese strains, we performed a population analysis using STRUCTURE software [28]. For this analysis, we used the sequences of 51 Nepalese strains and 1,209 reference strains, including strains from hpAfrica2, hpAfrica1, hpNEAfrica, hpEurope, hpSahul, hpAsia2, hspMaori, hspAmerind, and hspEAsia populations, that were deposited in the pubMLST database (60, 181, 61, 566, 49, 17, 80, 18, and 177 strains, respectively). STRUCTURE software performs MCMC simulations to classify individuals into a given number of populations (K). For a given K, STRUCTURE determines K population components and represents them by K colors, using one color to represent one population component. We performed the STRUCTURE analysis by setting Ks from 7 to 15 and executing simulations five times for each K. Fig 3 shows the results of K = 15, whose posterior probability is the best of the five runs (the most probable results). Each vertical line of the bar chart represents one strain, and the colors of a line indicate populations to which the strain may belong. The lengths of the colors in a line are proportional to the probabilities that the strain belongs to each population.

In the result of the K = 15 analysis, the Nepalese strains showed the most color commonality with hpAsia2 strains but differed from others that represents a population component specific to these strains (red bars marked with stars in Fig 3A). Fig 3C and and 3D are a magnification of the Nepalese strains. The bars were aligned from left to right in descending order of red components (Fig 3C) and navy blue and dark green components (Fig 3D). Interestingly, although Nepalese strains had basic similarity with hpAsia2 strains (Fig 3B), they also shared components with hpEurope strains (dark green bars in Fig 3C) that have not been found in hpAsia2. Furthermore, the Nepalese strains had a specific component that was very rarely found in other populations (navy blue bars). We aligned the data taken from PubMLST (1,209 reference strains) in descending order of navy blue components. The strain hk215 from an ethnic Chinese (Sino-Tibetan), strain kaz3172 from an ethnic Kazakh, and strain ku266 from an ethnic European (Germany) [11], were the top three ranking strains that shared this specific component found in Nepal strains (image not shown). The recombination genotype strains in Table 4 are marked with white dots in Fig 3D. As this figure shows, the recombination genotype was frequent in groups II and III. In the bar of East-Asian-type-*cagA* strains marked with a black triangle in Fig 3D, two strains showed shared components with hpEAsia strains (dark yellow bars). Therefore, by a detailed population structure analysis, Nepalese strains are distinguishable as belonging to three groups: hpAsia2, a recombination of hpAsia2 and typical Nepal, and a recombination of hpAsia2 and hpEurope. Only Nepalese strains that shared hpEurope were associated with greater inflammation in the antrum than the strains from the Nepalese specific population (mean [median]; 1.90 [2] vs. 1.50 [1.5]; P = 0.05).

### Nucleotide sequencing

Nucleotide sequence data reported are available under the DDBJ accession numbers LC042609 to LC043067.

## Discussion

We found that the prevalence of *H*. *pylori* infection, assessed using a combination of three diagnostic tests, in Kathmandu, Nepal was 38.4%, similar to the data from India [29], but much lower than Bhutan [19], or other neighboring countries. Natural springs as a source of drinking water were associated with an increased risk of infection. Therefore, consistent with several previous studies [30,31], *H*. *pylori* can plausibly survive and contaminate the local water supplies.

Several studies have examined the prevalence of *H*. *pylori* in Nepal (Table 5); however results from these studies have varied (16.3–70.5%) [17,18]. Among seven studies, six used histological examination for diagnosis [17,32–36]. One study reported low infection rates (16.3%) [17]; however, five studies reported high infection rates (33.9–68%) [32–36]. These differences might be due to the different evaluation criteria adopted by the different studies, as well as sample size, and use of different pathologists to read histological results. Six studies included patients with dyspepsia [17,32–36], whereas another study used the general population [18]. The *H*. *pylori* infection rate determined by a stool antigen test showed the highest results (70.5%) [18]; however that study determined the prevalence of a minor indigenous ethnic group in the eastern Himalaya. Therefore the high infection rate in this area cannot be generalized to all of Nepal.

**Table 5:** Summary previous *H*. *pylori* prevalence studies in Nepal.

First Author (ref.)	study period	Area	subject	n	Average age (range)	Test	Positive rate
Shrestha R [36]	2011–2013	Lalitpur	dyspeptic	228	44.7 (16–87)	Histology	68% (155/228)
Thapa R [17]	2011–2012	Kathmandu	dyspeptic	80	20–80	Histology	16.25% (13/80)
Shrestha S [32]	2011–2012	Kathmandu	dyspeptic	319	42.92 (15–87)	Histology	50.4% (161/319)
Sherpa TW [18]	no information	upper khumbu	general population	383	no information	Stool antigen	70.5%(270/383)
Dhakhwa R [33]	2011	Kathmandu	dyspeptic	200	41.5 (18–79)	Histology	44.0% (88/200)
Matsuhisa T [34]	2004–2005	Kathmandu	dyspeptic	309	no information	Histology	54% (167/309)
Makaju RK [35]	2004–2005	Kathmandu	dyspeptic	224	no information	Histology	33.9% (76/224)

To our knowledge, this report is the first to reveal the virulence factors of *H*. *pylori* in Nepal. We found that most of the pre-EPIYA and *cagA* segments in the Nepalese strains were considered to be non-deletion types and Western-type-*cagA*, respectively. Compared with individuals harboring Western-type-*cagA* strains containing EPIYA-C segments, those infected with East-Asian-type-*cagA* strains containing EPIYA-D segments have been reported to have an increased risk of peptic ulcer or gastric cancer [25,37]. These results may explain the “Asian paradox” phenomenon in Nepal, similar to *H*. *pylori* isolates from India and Pakistan [38–40], where the *H*. *pylori* infection rate among the Nepalese population is high but risk of gastric cancer is low, suggesting that the Western-type-*cagA* is likely to be associated with the reduced risk of gastric cancer in these populations. Histology results in this study were unusual, in that Western-type-*cagA* was more virulent than East-Asian-type-*cagA*, which might be explained partly by the fact that all East-Asian-type-*cagA* strains included the recombination genotype. Recombination is known to be extremely frequent in *H*. *pylori* [41], which is related to adaptation during chronic colonization [42]. The recombination process could affect protein expression in a large number of genes encoding surface structures (lipopolysaccharide, flagella, outer membrane proteins, beta-lactamases), restriction modification genes, and hypothetical ORFs [43]. This study also confirmed that all strains from DU, GU, and gastric cancer cases were associated with the Western-type-*cagA* and pre-EPIYA non-deletion type (non-recombination genotype) infection. The Asian paradox phenomenon might also be explained by the high proportion of m2 genotypes in Nepal, which is similar to other countries such as Thailand [44] and Bangladesh [45] that have low incidences of gastric cancer. However, we also considered that, although gastric carcinogenesis might be influenced by *H*. *pylori* virulence factors, the host’s genetic and environmental factors should also be important in determining the risk of gastric cancer.

The predominance of *vacA* s1a in the Nepalese strains is similar to a previous report that showed that the s1a subtype was present in almost all strains from South Asia, Northern-Eastern Europe, and also Malaysia [46]. Nepalese isolates with the *vacA* m genotype were closely related to each other, as well as the strains with the *vacA* m1c allele from India and Bangladesh. These results were confirmed by a population structure analysis, which showed that Nepalese strains are similar to hpAsia2 strains in accordance with human genetic analyses [16]. The hpAsia2 population was initially defined based on isolates from Ladakh in Northern India, which represents a west/middle Asia population [47]. The recombination of hpAsia2 and hpEurope in the Nepalese strains also confirmed what was found in the previous study in India: that most strains initially belonged to hpAsia2 [11], while some strains belonged to hpEurope [48]. It remains unclear what other populations share this typical Nepalese component, and whether this component originated in Germany and became an ancestor of *H*. *pylori* in ethnic Chinese or whether this is the result of mixing between more than two populations. It has previously been suggested that hpEurope can be divided into Ancestral European 1 (AE1) and Ancestral European 2 (AE2). AE1 originated in Central Asia, because it shares phylogenetic signals with isolates from Estonia, Finland, and Ladakh in India. It is not clear which population arrived first, but AE1 has a higher frequency in Northern Europe, while AE2 is more common in Southern Europe. A previous study also found a mixed AE1 and AE2 ancestry in the near East and in the Indian subcontinent [48]. Kathmandu valley, a region located in the east central hills of present-day Nepal, has witnessed several different waves of migrations, therefore it is not surprising that the indigenous inhabitants, the Newars, are postulated to be a mixture of Austro-Asiatic, Dravidian, IndoMongoloid, and Aryan origins. The earliest rulers of the Kathmandu Valley were the Tibeto-Burman Kiratas (4^th^ century C.E). The Kiratas were subsequently replaced by the Licchavi Dynasty (400–800 C.E.), a Hindu group that migrated from northern India. By the 13th century C.E., they were replaced by the Malla Dynasty [16]. Nepal was never colonized by Europeans, therefore the possible sources of genetic heterogeneity in the Nepalese *H*. *pylori* isolates is likely from a centuries-old importation. Further study is necessary to elucidate these more virulent of Nepalese strains, which share hpEurope disproportionately. However, this association can be indirectly explained by the fact that ASR of gastric cancer in the European continent are higher than in the South-Central Asia region (9.4 and 6.7/100,000, respectively).

First, the small number of strains included is a limitation in this study. Further studies with an increased number of samples, balanced for each diagnosis, will be necessary to better understand the association of virulence factors and clinical outcomes in Nepal. Second, we obtained samples from a hospital in Kathmandu, the capital and largest metropolis in Nepal. The physical and cultural landscape varies by area in Nepal. Therefore, our results cannot be generalized across all of Nepal.

## Conclusion

We found that most Nepalese strains were of the characteristic South Asian genotypes, Western-type-*cagA* with no deletion, s1a, m1c, and a high proportion of m2. However many Nepalese strains were *H*. *pylori* recombinants that showed genetic features of South Asian and East Asian genotypes, which a less virulent than specific South Asian genotypes. In addition, although the population structure confirmed that most Nepalese strains belonged to hpAsia2, some of them shared components found in hpEurope and typical Nepal strains. Therefore, these results may support the hypothesis that the ancestor roots of Kathmandu`s peoples not only connected with people from India alone.
